# Nonenteric Adenoviruses Associated with Gastroenteritis in Hospitalized Children

**DOI:** 10.1128/spectrum.00300-21

**Published:** 2021-07-28

**Authors:** Maria Antonia De Francesco, Giovanni Lorenzin, Antonella Meini, Richard Fabian Schumacher, Arnaldo Caruso

**Affiliations:** a Institute of Microbiology, Department of Molecular and Translational Medicine, University of Brescia–Spedali Civili, Brescia, Italy; b Pediatrics Clinic, Children’s Hospital, ASST Spedali Civili of Brescia, Brescia, Italy; c Haemato-Oncology and BMT Unit, Children’s Hospital, ASST Spedali Civili, Brescia, Italy; Memorial Sloan Kettering Cancer Center

**Keywords:** adenovirus, gastroenteritis, immunocompromised hosts

## Abstract

The object of this study was to investigate the frequency of human adenovirus (HAdV) infections in hospitalized pediatric patients. Stool samples were collected during a 1-year period (February 2018 to January 2019). HAdV was detected by a broad-range PCR and genotyped by sequencing and phylogenetic analysis. Demographic characteristics and detailed clinical information were analyzed for each patient. HAdV was detected in 7.1% of stool samples (34/476). Among these patients, 23.5% were coinfected with other enteric viral or bacterial pathogens. The majority (85.2%) of HAdV positives were detected in children of <5 years of age. Two HAdV species (B and C) with three types were identified in this study population. HAdV species F was not detected. Genetic analysis shows that the isolates circulating in our region present high diversity and do not exhibit clonal expansion. The presence of nonenteric HAdV in subjects with gastrointestinal symptoms and in immunocompromised patients has already been reported by different studies and underlines the need to develop routine molecular assays that have wide reactivity for most types of adenovirus in order to obtain an optimal tool for their rapid and accurate diagnosis.

**IMPORTANCE** Gastroenteritis is the second leading cause of death among infants and children worldwide. Our study shows that adenovirus types other than 40 and 41 might be related to acute gastroenteritis. Therefore, a novel approach using diagnostic methods able to detect all adenovirus types is desirable in order to overcome the limitations of the current techniques.

## INTRODUCTION

Acute gastroenteritis (AGE) is a leading cause of morbidity and mortality in infants and young children worldwide, particularly in developing countries (1). Viruses are the most important etiologic agents, with rotavirus group A (RVA) being the most frequent cause of AGE (2). Following the introduction of RVA vaccines in 2006, other enteric viruses have occurred with higher prevalence (3, 4) such as human adenoviruses (HAdV) (5–8).

Human adenovirus belongs to the Mastadenovirus genus and is currently grouped into nine subgroups (A to I), further divided into more than 100 genotypes (https://talk.ictvonline.org/taxonomy and http://hadvwg.gmu.edu) (9). Among these, species F (types 40 and 41) is associated with infection of the gastrointestinal tract and is responsible for up to 20% of episodes of diarrhea around the world (10–13). However, other types belonging to different species, including HAdV-A, HAdV-B, HAdV-C, HAdV-D, and HAdV-G, have also been detected in children with gastroenteritis (9, 14–18).

The aim of this study was to determine the prevalence of adenovirus in a pediatric population with AGE from February 2018 to January 2019 and to perform genotyping of the detected HAdV strains in order to obtain information about their molecular epidemiology.

Molecular typing is of particular importance in detecting other genotypes rather than the “classical” enteric adenoviruses that play a leading role in a significant number of gastroenteritis cases seen in immunocompromised children.

## RESULTS

Adenovirus was detected in 34 (7.1%) of 476 fecal specimens. The mean and median ages of HAdV-positive patients were 2.6 years (range, 11 days to 17 years) and 1 year, respectively. Patient characteristics are summarized in Table 1. Gastrointestinal symptoms were present in 24 patients (70.5%); six (17.6%) had fever, associated in two cases with respiratory infections, and for four patients, clinical symptoms were not available. Acute enteritis diagnosis was the reason for hospitalization for 13 patients (13/34; 38.2%) (Table 1). Among the 34 HAdV-positive patients, 53% (18/34) were male and 47% (16/34) were female (1.1:1). The proportions of HAdV infection found in male and female groups were similar at 7.4% (18/243) and 6.8% (16/233), respectively, and this difference was not statistically significant (*P = *0.8) (Table 1). In the cohort of children aged between 0 and 2 years, 10.8% (24/222) were positive for HAdV; in the group of children aged between >2 and 5 years, 4.2% (5/118) were positive; and in children aged >5 years, 3.6% (5/136) were positive cases. HAdV infection was significantly more common in patients aged <2 years than in older age groups (*P = *0.006), confirming that this virus is a particularly important pathogen for gastrointestinal infections in infants and young children (Table 1).

**TABLE 1:** Adenovirus-positive patients’ characteristics and typing results

Patient	Age	Gender^*a*^	Main symptom(s)^*b*^	Underlying disease and/or main reason for hospitalization^*c*^	Coinfection^*d*^	Genotype
1	3 yrs	F	Diarrhea	Enteritis	Rotavirus G1[P8]	C2
2	2 yrs	M	NA	Epilepsy	None	C1
3	11 days	F	NA	Prematurity	None	C1
4	3 mo	M	Fever/diarrhea	Congenital rubella	None	C2
5	2 yrs	M	Diarrhea	Intussusception	None	C2
6	1 mo	M	Fever/bloody stools	Prematurity	None	C2
7	1 yr	M	Fever	Acute pyelonephritis	Campylobacter species	C2
8	3 yrs	F	Diarrhea/vomit	Acute adenomesenteritis	None	C2
9	1 yr	F	Fever/diarrhea	Acute enteritis	C. difficile	C2
10	1 yr	F	Fever	Pneumonia	PIV	C2
11	8 yrs	M	Fever	Pharyngotonsillitis	None	C2
12	1 mo	F	Diarrhea	Prematurity	None	C2
13	1 yr	M	Fever	Kawasaki syndrome	None	C1
14	4 yrs	F	Fever	Cystic fibrosis	None	C1
15	1 yr	F	Diarrhea	HSCT	None	C1
16	3 mo	F	Diarrhea/vomit	Surgery for biliary atresia	None	C1
17	1 yr	M	Fever	Infectious mononucleosis	None	C1
18	14 yrs	F	Diarrhea	Psychosomatic disorders	Campylobacter species	C1
19	7 yrs	M	Diarrhea	NA	EPEC	C1
20	1 yr	M	NA	HSCT	None	C1
21	1 yr	M	Diarrhea	Acute enteritis	None	C1
22	1 yr	M	Diarrhea	Acute enteritis	None	C2
23	4 yrs	M	Diarrhea/vomit	Acute enteritis	None	B3
24	1 yr	M	Diarrhea/vomit	Acute enteritis	None	C2
25	17 yrs	F	Diarrhea	SCID	None	C2
26	6 mo	M	Diarrhea	Yolk Sac Tumor	None	C2
27	1 yr	M	Diarrhea	Acute enteritis	None	C2
28	1 yr	F	Diarrhea/vomit	Acute enteritis	None	C2
29	1 yr	F	Diarrhea/vomit	Acute enteritis	None	C2
30	8 yrs	M	NA	NA	None	B3
31	5 yrs	F	Diarrhea	Enteritis	None	C1
32	1 yr	M	Diarrhea	Enteritis	None	C1
33	2 yrs	F	Diarrhea	Enteritis	Coxsackie A22	C1
34	3 mo	F	Diarrhea	Enteritis	Rotavirus G1[P8]	B3

a M, male; F, female.

b NA, not available.

c SCID, severe combined immunodeficiency; HSCT, hematopoietic stem cell transplant.

d EPEC, enteropathogenic Escherichia coli; PIV, parainfluenza virus.

HAdVs were detected almost throughout the, year with the highest prevalence between April and June (26/34, corresponding to 76.4% of all positive patients; *P* < 0.0001).

HAdV was the only pathogen identified in 26 patients (26/34; 76.5%), while in 8/34 (23.5%) specimens, other viruses and different bacterial pathogens were detected. In particular, we found coinfection with rotavirus (2 cases), parainfluenza virus (1 case), coxsackie virus (1 case), Campylobacter species (2 cases), enteropathogenic Escherichia coli (1 case), and Clostridium difficile (1 case).

All isolates were successfully sequenced. A phylogenetic tree according to partial hexon gene (482-bp fragment) sequencing was constructed to identify HAdV species and genotypes. Over the 1-year study period, species C (31/34; 91.2%) and B (3/34; 8.8%) were detected, and three genotypes were identified. Of the three genotypes identified, HAdV-C2 (17/34; 50%) was the prevalent genotype, followed by HAdV-C1 (14/34; 41.2%) and HAdV-B3 (3/34; 8.8%). The genetic trees include the strains sequenced in this study along with prototype sequences.

**FIG 1:**
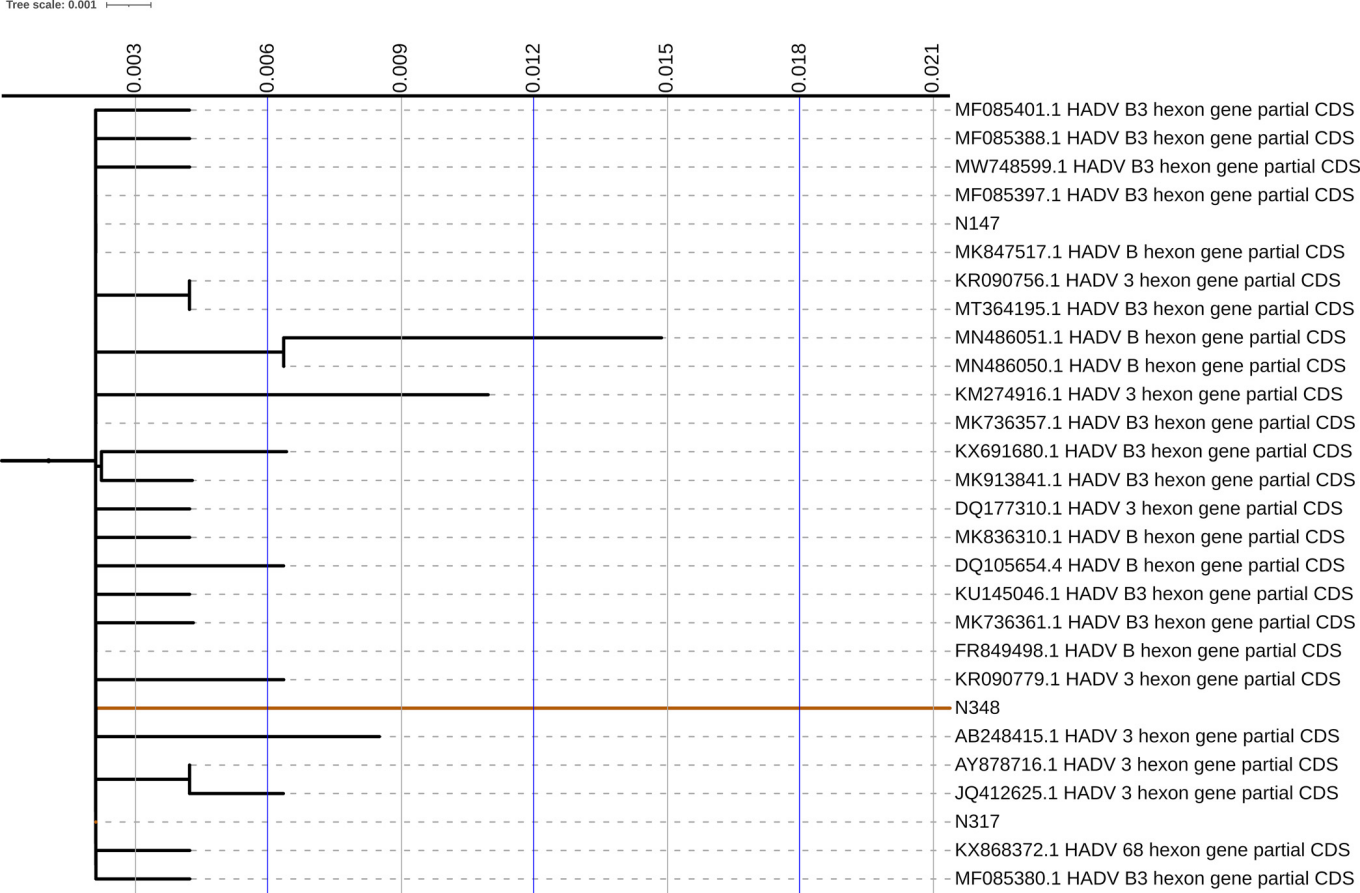
Phylogenetic trees of partial nucleotide sequences of the hexon gene. Gene sequences of hexons from adenovirus type 3 are presented. Sequences derived from this study are identified by the wording “N” before the number of the isolate. GenBank sequences of the corresponding HAdV species prototypes are identified by their accession number, species, and type. The neighbor-joining tree was generated by using IQ-TREE and implementing a bootstrap test of 5,000 replicates.

**FIG 2:**
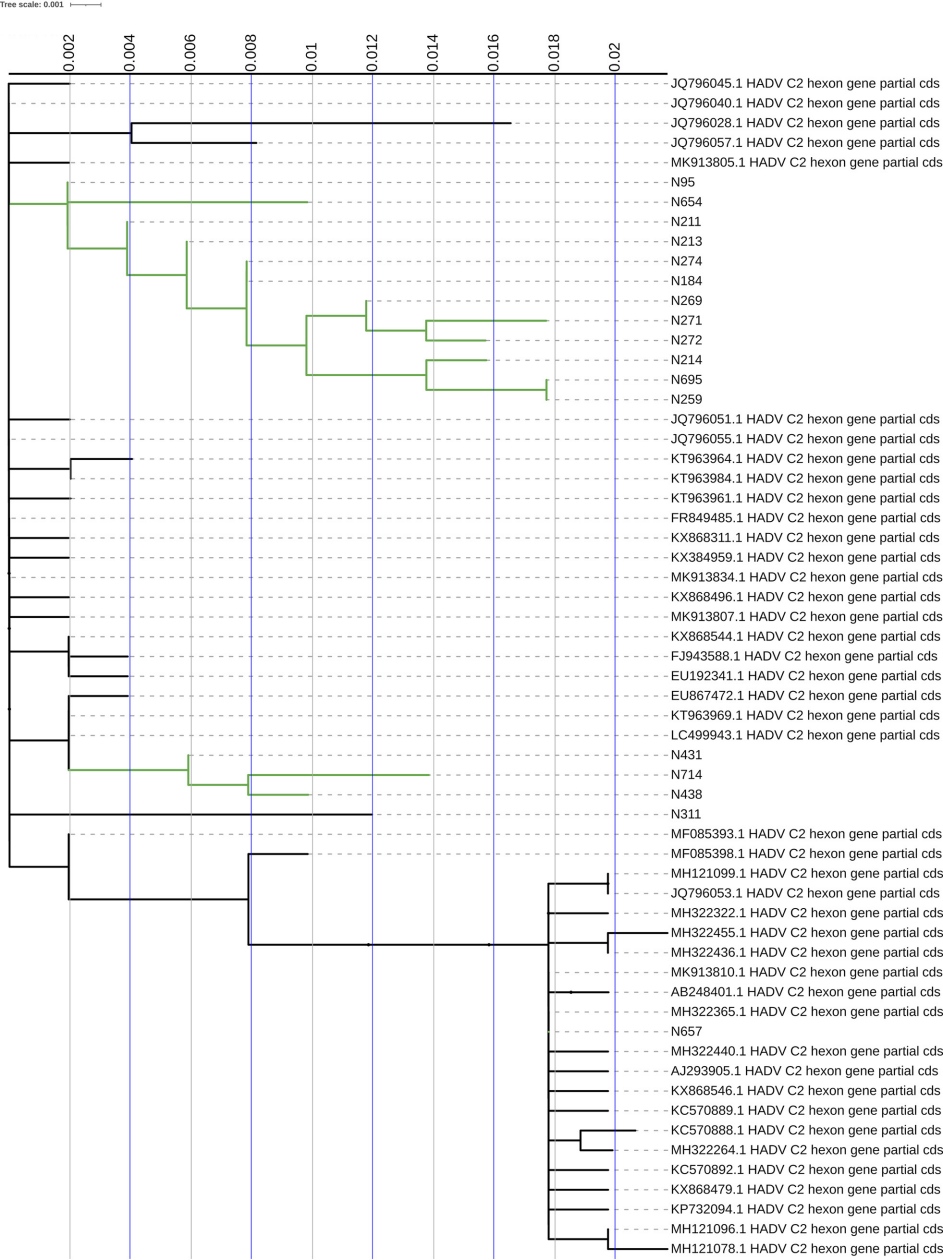
Phylogenetic trees of partial nucleotide sequences of the hexon gene. Gene sequences of hexons from adenovirus type 2 are presented. Sequences derived from this study are identified by the wording “N” before the number of the isolate. GenBank sequences of the corresponding HAdV species prototypes are identified by their accession number, species. and type. The neighbor-joining tree was generated by using IQ-TREE and implementing a bootstrap test of 5,000 replicates.

Regarding species B type 3, the three genotyped isolates form a small isolated clade; no neighboring sequence has European geographical origin. No particular mutations were found, and these sequences fall perfectly among those selected to represent the variability of subtype 3 (Fig. 1). For species C type 2 it can be noted that most of the genotyped isolates form a large clade; as hypothesized, these strains, which are possibly responsible for gastroenteritis, differ from those chosen in the creation of the genotyping database that come from upper respiratory isolates. There is no geographic relationship between these either (United States or China) (Fig. 2).

**FIG 3:**
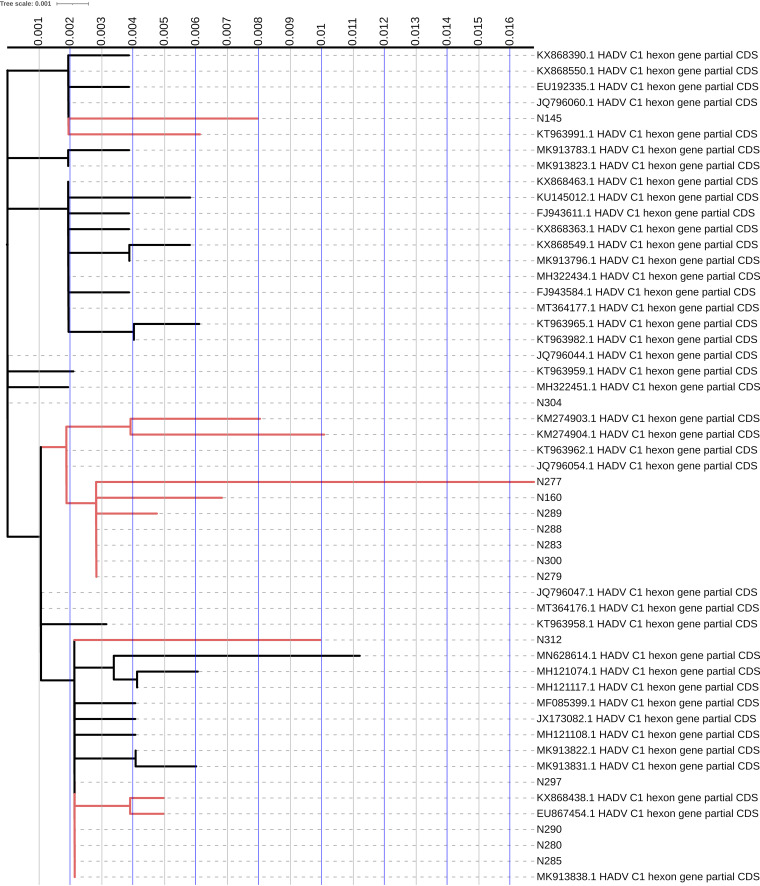
Phylogenetic trees of partial nucleotide sequences of the hexon gene. Gene sequences of hexons from adenovirus type 1 are presented. Sequences derived from this study are identified by the wording “N” before the number of the isolate. GenBank sequences of the corresponding HAdV species prototypes are identified by their accession number, species. and type. The neighbor-joining tree was generated by using IQ-TREE and implementing a bootstrap test of 5,000 replicates.

Regarding species C type 1, in this case we also have a correct positioning within the subtree chosen for genotyping. C1 forms a single well-delineated clade that is phylogenetically distant geographically from neighboring sequences (Fig. 3). There is a certain distance between the sequenced isolates; this presupposes an exclusion of clonal expansion of the strains but suggests that the different isolates evolved differently.

## DISCUSSION

In this study, we identified an overall HAdV infection rate of 7.1%, a percentage similar to the 6.8% we described in a previous publication (19) and to those reported by other studies from Thailand (14), Japan (20), China (21), and India (11).

No association was found between gender and adenovirus infection, a finding already reported in different studies (19, 21). We confirmed a higher incidence of HAdV infection in children aged <2 years (22), which is probably related to an increase of neutralizing antibodies with age, which prevent reinfection with the same serotype and thus are responsible for a lower frequency of infection in the older age groups.

We observed a seasonal distribution with a significant prevalence of HAdV-positive cases in spring (from April to June). Some studies have reported a seasonal distribution with a higher incidence of HAdV infection during the warm and/or the rainy season (11, 23), while others did not find any seasonal association (5, 24). These contradictory results might be due to the weather patterns of different countries.

In 8/34 (23.5%) patients, we found coinfection with different viruses and bacteria. A dual infection can be explained by the fact that some microorganisms continue to be excreted for some time after the initial acute episode, while another pathogen starts a new acute infection. However, the clinical relevance of the pathogens identified with the gastroenteritis observed was difficult to establish in coinfected patients (25).

Molecular typing based on partial hexon gene sequencing identified a prevalence of two species of adenovirus (C, 31/34 [91.2%], and B, 3/34 [8.8%]), with three different types belonging to these species. Genetic analysis show that the isolates circulating in our region were not related to European strains. In particular, for HAdV species C type 2, our isolates were phylogenetically distant from the species C type 2 responsible for respiratory infections. HAdV species F types 40 and 41, which have tropism for the gastrointestinal tract and are among the most common adenoviruses isolated from patients with gastroenteritis, were not detected in this study.

Because the detection of adenoviruses different from the “enteric” types in stool could be due to the prolonged shedding in feces after previous infections in other organs (9, 23), we retrospectively analyzed the clinical picture of our patients. Thirteen patients had a diagnosis of acute enteritis. Six patients presented with an immunocompromising condition, as follows: two were premature infants (26), two received a hematopoietic stem cell transplant, and one patient had a yolk sac tumor with a progressive lymphopenia (1.6 × 10^3^ cells per mm^3^) and was subjected to the first cycle of bleomycin, etoposide, and cisplatin (BEP) treatment; the last patient had a severe combined immunodeficiency. In this category of patients, represented by subjects with compromised immune responses, in particular allogeneic hematopoietic stem cell transplant recipients, it was already shown that more than half of adenovirus infections (27–29) are associated with adenovirus species C types 1 and 2, the species found to be prevelent in this study.

All of the patients except one had throat swabs negative for adenovirus, and only two patients had a clinical diagnosis of respiratory infections, suggesting a possible involvement of the adenovirus species found in the stool.

Three patients had different clinical conditions, such as intussusception, biliary atresia, and Kawasaki disease, which have been associated with adenovirus (30–35).

However, the number studies worldwide that report an association between nonenteric adenoviruses and gastroenteritis has increased (15, 25, 36, 37).

In fact, HAdV-B3 has been associated with diarrhea in infants and children (36); HAdV-C1, HAdV-C2, and HAdV-C5 are genotypes that are frequently detected in patients with AGE (36). Furthermore, HAdV-A12, HAdV-A18, HAdV-A31, and HAdV-G52 have been associated with enteric symptoms (18, 25). Similarly to these studies, our findings also contribute to the growing evidence for a potential role of so-called “nonenteric.”

So far, since most of the commercial assays detect only adenovirus 40 and 41, it is advisable that new rapid and reliable detection methods should be developed for all HAdV types. This should provide more accurate information about the true burden of gastroenteritis caused by different adenovirus types, especially in immunocompromised patients, who are at higher risk to develop adenovirus invasive disease and complications, as already reported (38, 39).

## MATERIALS AND METHODS

Between February 2018 and January 2019, a total of 476 stool specimens were collected from 476 children with AGE symptoms, aged from 11 days to 17 years old and admitted to the Children’s Hospital, ASST Spedali Civili, Brescia, Italy. Acute gastroenteritis symptoms included fever, abdominal pain, vomiting, bloating, and diarrhea. The specimens were tested by routine assays for enteric bacteria and viruses and then stored in phosphate-buffered saline at 10% suspensions at −80°C until use. All molecular assays were performed retrospectively on fecal samples collected during the study period. Viral nucleic acid was extracted from 10% clarified stool suspensions prepared in phosphate-buffered saline by using the QIAamp viral RNA minikit (Qiagen, Milan, Italy) according to the manufacturers’ instructions. Clinical, bacteriological, and virological data were extracted retrospectively from electronic reports and were anonymized before being included in the study database. This study did not require ethical approval, as it did not involve a prospective evaluation. The samples were left over from routine investigation and were used anonymously. Formal consent was not required due to the retrospective format of the study.

Adenoviruses were detected by a real-time PCR with primary and proved with nested PCR confirming positive samples as previously described (40, 41). PCR products were visualized on a 1.5% agarose gel, stained with GelRed nucleic acid stain (D.B.A. Italia s.r.l. Milan, Italy) under a blue-light transilluminator. The species, group, and serotype were assigned on a genetic basis using the sequences collected in the NCBI RefSeq database and the associated “neighbor nucleotides” database present on the NCBI nucleotide archive as a comparison data set. The full sequences of 652 HAdV genomes were selected, and the data set was completed with the genomic sequences under study. The data set was then edited manually by removing redundant sequences from the data set; those not geographically related and/or from different studies were included. For the alignment of the sequences, we used the latest available version of MAFFT multiple-sequence alignment software (MAFFT v7.475, method L-INS), first on the entire data set and then, after careful trimming of the areas not included in the alignment and splitting of the sequences based on types, on the final data sets (42). The phylogenetic analysis was first performed by choosing the best nucleotide substitution model, and a maximum-likelihood tree was subsequently generated, to which a bootstrap of 5,000 repetitions was applied (IQ-TREE v1.6.12) (43). A total of three trees were generated, representing typing and genetic relation between isolates. Differences in proportions were tested using the χ^2^ test. A *P* value of <0.05 was considered statistically significant.

### Data availability.

The GenBank accession numbers for the sequences of the hexon genes of the study strains are MZ395500 to MZ395533.
